# Supplementary material for: Differential Upregulation of Th1/Th17-Associated Proteins and PD-L1 in Granulomatous Mycosis Fungoides
Source: Cells. 2024 Feb 27;13(5):419. doi: 10.3390/cells13050419 (PMC10931377; doi:10.3390/cells13050419)
Supplement: Supplementary file 1 [file cells-13-00419-s001.zip › cells-2810280-supplementary.pdf]

Table S1. Antibodies and dilutions of immunohistochemical markers used in the diagnostic assessment of patients with granulomatous mycosis fungoides (GMF) and mycosis fungoides with large cell transformation (MFLCT).

**Immunohistochemical Marker**	**Conditions**		
	**Antibody Clone**	**Catalogue#**	**Dilutions**
**CD1a**	MTB1	PA0235	RTU
**CD2**	AB75	NCL-L-CD2-271	1:75
**CD3**	LN10	PA0122	RTU
**CD4**	SP35	104R-16	1:25
**CD5**	4C7	MS-393-S	1:20
**CD7**	4C7	PA0168	RTU
**CD8**	LP15	NCL-L-CD7-580	1:100
**CD20**	4B11	MS-47-S	RTU
**CD25**	L26	PA0200	RTU
**CD30**	4C9	PA0305	RTU
**EBER**	Ber-H2	790-4858	RTU
**TCRBF1**	8A3	TCR1151	1:100
**TCRG**	H41	sc-100289	1:150

RTU, ready to use.

Table S2. Number of events according different variables for the cohort.

	**Cohort**
Clinical Stage	
Stage 1	5/13
Stage 2	11/16
Stage 4	12/13
Unknown	2/7
Group	
GMF	12/28
MFLCT	18/21
%ROR꩜T	
<10%	17/21
≥10%	13/28
%Foxp3	
<10%	10/12
≥10%	20/37

GMF: granulomatous mycosis fungoides; MFLCT: mycosis fungoides with large cell transformation.
